# Knowledge, Attitude, and Behavior of Medical Students with Regard to Concussions: A Cross-Sectional Study

**DOI:** 10.7759/cureus.47112

**Published:** 2023-10-16

**Authors:** Sami F Almalki, Ossama M Zakaria, Abdulelah S Almousa, Muhannad M Alwadany

**Affiliations:** 1 Neurosurgery, King Faisal University, Hofuf, SAU; 2 General Practice, King Faisal University, Hofuf, SAU

**Keywords:** observational cross-sectional study, saudi arabia, medical students, knowledge and perception, medical education, brain concussion, traumatic brain injury

## Abstract

Background: Concussions, categorized as mild traumatic brain injuries, result from traumatic events and present a significant concern within the field of traumatic brain injuries. Understanding the multifaceted pathophysiology of concussions, their diverse symptomatology, and their appropriate management strategies is crucial for healthcare professionals. This study explores the knowledge, attitudes, and behaviors of medical students at King Faisal University in the Eastern Province of Saudi Arabia regarding concussions.

Methods: A cross-sectional study design was employed to assess a diverse group of medical students at King Faisal University in the Eastern Province of Saudi Arabia. Participants were surveyed using a questionnaire covering socio-demographic information, knowledge assessment, attitude assessment, and behavior assessment.

Results: Of the 315 participants, 68.3% demonstrated good knowledge about concussions. Participants generally recognized concussions as a type of traumatic brain injury (68.9%) and believed it was necessary to report concussion symptoms to a doctor (80.3%). However, certain misconceptions existed, such as the belief that all patients with concussion should rest for seven days (31.7%). Participants primarily obtained information from teachers (100%) and the internet and social media (81.6%).

Conclusion: While medical students at King Faisal University in the Eastern Province of Saudi Arabia generally exhibited good knowledge about concussions, specific knowledge gaps and misconceptions were seen to exist. To ensure comprehensive understanding and promote appropriate management, continuous education, and awareness campaigns are essential, with healthcare providers playing a pivotal role in knowledge dissemination.

## Introduction

Concussions, often described as transient disturbances in neurological function resulting from traumatic events, are a significant concern within the realm of traumatic brain injuries. These injuries encompass a wide spectrum, ranging from mild to severe, with concussions representing a subset categorized as mild traumatic brain injuries. While concussions can result from direct head impacts, such as collisions, falls, or attacks, they can also occur due to indirect injuries, where rapid head movement stems from trauma in other parts of the body [1-3].

Understanding the pathophysiology of concussions is complex, involving mechanisms such as shearing forces, changes in head movement, axonal injury, and neurotransmitter release caused by ion channel depolarization. Additionally, disruptions in glucose levels, altered energy utilization, mitochondrial dysfunction, and reduced cerebral blood flow contribute to the intricate nature of concussion pathophysiology [4,5].

Concussions manifest through a diverse range of symptoms, broadly categorized into four groups. These include emotional disturbances, cognitive impairments, physical symptoms, and disruptions in sleep patterns. Notably, loss of consciousness is observed in a minority of cases, emphasizing the heterogeneity of concussion presentations [1,2].

Diagnosing concussions relies on a multifaceted approach that includes assessing patient history, injury type, symptom characteristics, onset timing, severity, and duration. Physical, neurological, cognitive, and emotional evaluations are essential components of the diagnostic process. Various tools, such as the Sport Concussion Assessment Tool 5, aid in this assessment. It's important to note, however, that the diagnosis of concussion may not provide insight into its precise underlying pathological mechanism [6,7].

Concussion management strategies are highly individualized, with the primary aim of providing symptomatic relief. Initial treatment typically involves rest recommendations to limit physical and cognitive activities for the initial 24-48 hours post injury [8]. Gradual resumption of activities is advised, with careful monitoring for symptom recurrence or worsening and reduction of activity as needed [8-10].

By studying the knowledge, attitude, and behavior of medical students in the Eastern Province of Saudi Arabia regarding concussions, we seek to address a critical gap in understanding how future healthcare professionals perceive and respond to this common yet complex injury. This research is vital for the development of educational and preventive strategies to enhance concussion management within the healthcare community.

## Materials and methods

Study design

This investigation employed a cross-sectional study design to comprehensively explore the knowledge, attitudes, and behaviors of medical students at King Faisal University in the Eastern Province of Saudi Arabia regarding concussions at a specific point in time.

Study population

The study was conducted at King Faisal University in the Eastern Province of Saudi Arabia. Inclusion criteria encompassed individuals enrolled as medical students at King Faisal University, with representation from different academic years, genders, and nationalities. Exclusion criteria encompassed individuals who did not meet these inclusion criteria or chose not to participate.

Survey questionnaire

The survey questionnaire, meticulously designed based on validated tools from prior research [9], was aimed at assessing the knowledge, attitudes, and behaviors of participants concerning concussions. This questionnaire included multiple sections, such as socio-demographic information, knowledge assessment, attitude assessment, and behavior assessment. Each question in the questionnaire was scored, with correct responses earning 2 points and incorrect answers receiving 0 points. Participants who scored 75% or more correct answers (equivalent to 27 points out of 36) were considered to possess good knowledge, attitudes, and behaviors regarding concussions.

Prior to the main data collection, a pilot study was conducted to validate the survey questionnaire. A total of 20 students from King Faisal University, representing various academic years, participated in the pilot study. The questionnaire was reviewed by experts in the fields of emergency medicine and neurosurgery to assess its face and content validity. These experts provided valuable feedback and recommendations, which were carefully considered and incorporated into the final version of the questionnaire. Furthermore, the internal consistency of the questionnaire was assessed using Cronbach's alpha, with a resulting value of 0.85. This demonstrated a high level of consistency and reliability among the questionnaire items. The validated questionnaire was then used in the main data collection phase to maintain the reliability and validity of the study.

Sample size calculation

The sample size for this study was determined using the OpenEpi tool (Centers for Disease Control and Prevention, Atlanta, Georgia, United States). The population size, defined as 1300, represented the total number of medical students at King Faisal University in the Eastern Province of Saudi Arabia. A hypothesized frequency of the outcome factor, which in this case is defined as "good knowledge about concussion," was set at 67%. This value was obtained from a previous study [11]. The margin of error was set at ±5%. The confidence limits were selected as 5% of 100. Since cluster surveys were not employed, a design effect was not applied. With a confidence level of 95%, the calculated sample size for this study was 270 participants.

Data collection

The survey for this study was distributed using an online platform to facilitate participant access and engagement. Online distribution was chosen to ensure the survey's convenience, making it readily available to a broad audience. Participants were provided with a link to the survey through email invitations and social media platforms. Convenience sampling was employed due to practical constraints, allowing us to gather responses from individuals who were readily accessible and willing to participate.

Data analysis

After verifying data for completeness and consistency, data analysis was carried out using the IBM Statistical Package for Social Sciences (SPSS) Statistics for Windows, Version 26.0 (Released 2019; IBM Corp., Armonk, New York, United States). Categorical variables were represented as percentages and frequency distributions. Continuous variables were expressed as means and standard deviations.

Ethical considerations

The study received approval from the Research Ethics Committee at King Faisal University (KFU-REC-2023-MAR-ETHICS663), ensuring strict adherence to ethical standards. All data were maintained in strict confidentiality and utilized exclusively for research purposes, with informed consent being obtained from all study participants.

## Results

Participant demographics

A total of 315 students from King Faisal University College of Medicine participated in this study. Of these participants, 178 (56.5%) were male and 137 (43.5%) were female. The age distribution revealed that 114 (36.2%) participants were in the 23-26 year age group, 106 (33.7%) were in the 20-22 year age group, and 95 (30.2%) were younger than 20 years old. In terms of their year of medical education, 58 (18.4%) were in the sixth year or interns, 56 (17.8%) in the fifth year, 54 (17.1%) in the fourth year, 52 (16.5%) in the third year, 50 (15.9%) in the second year, and 45 (14.3%) in the first year. Notably, all participants reported an average monthly income of less than 10,000 Saudi riyal (SAR) (Table 1).

**Table 1:** Socio-demographic characteristics of study participants (n=315) SAR: Saudi riyal

Variable	Categories	Frequency	Percent
Gender	Male	178	56.5
	Female	137	43.5
Age (in years)	<20	95	30.2
	20-22	106	33.7
	23-26	114	36.2
Year of medical education	First	45	14.3
	Second	50	15.9
	Third	52	16.5
	Fourth	54	17.1
	Fifth	56	17.8
	Sixth/intern	58	18.4
Average monthly income (SAR)	Less than 10,000	315	100
	10,000-14,999	0	0
	15,000-19,999	0	0
	20,000 or more	0	0

Knowledge of concussion

The participants' average knowledge score was 24.5 ± 10.2 out of 32, signifying variability in their understanding of concussions. Of the participants, 100 (31.7%) were categorized as having poor knowledge, while 215 (68.3%) were considered to have good knowledge. Table 2 summarizes the responses of participants to each question pertaining to the knowledge and awareness of concussion.

**Table 2:** Knowledge, attitudes, and behaviors regarding concussion among medical students

Question	Categories	N (%)
1. Have you ever heard about the term "concussion"?	Yes	315 (100)
	No	0 (0)
2. Do you know the term "traumatic brain injury"?	Yes	289 (91.7)
	No	26 (8.3)
3. Have you ever experienced a traumatic brain injury?	Yes	0 (0)
	No	315 (100)
4. Have you ever witnessed anybody experiencing a traumatic brain injury in front of you?	Yes	0 (0)
	No	315 (100)
5. Concussion is a type of traumatic brain injury.	True	217 (68.9)
	False	98 (31.1)
6. Concussions can only occur if the person facing a traumatic brain injury goes to a state of unconsciousness.	True	86 (27.3)
	False	229 (72.7)
7. Do concussion symptoms always occur immediately after the injury?	Yes	100 (31.7)
	No	215 (68.3)
8. Concussion symptoms are not very apparent and may remain unnoticed.	Yes	229 (72.7)
	No	86 (27.3)
9. A person without a direct head injury will not face concussions.	True	100 (31.7)
	False	215 (68.3)
10. Is it necessary to report the concussion symptoms to a doctor?	Yes	253 (80.3)
	No	41 (13)
	I am not sure	21 (6.7)
11. Concussions cannot occur years after the injury.	True	102 (32.4)
	False	213 (67.6)
12. Patients affected by concussion are advised to take rest and limit the _____ activities.	Physical	100 (31.7)
	Cognitive	0 (0)
	Both of them	215 (68.3)
	None of them	0 (0)
13. Patients are advised to resume their normal routine after__________.	The symptoms of concussion are gone	50 (15.9)
	One day of rest	17 (5.4)
	Three days of rest	21 (6.7)
	The doctor advises them	227 (72.1)
14. The treatment protocol for concussion is straightforward and defined.	True	55 (17.5)
	False	260 (82.5)
15. Should all patients affected by concussions take rest for seven days?	Agree	100 (31.7)
	Disagree	215 (68.3)
16. Is it necessary to perform CT scans of all the patients facing concussions after a traumatic brain injury?	Agree	92 (29.2)
	Disagree	223 (70.8)
17. Concussion management is based on symptomatic relief.	Agree	269 (85.4)
	Disagree	46 (14.6)
18. Do you think you have sufficient awareness about concussions?	Yes	164 (52.1)
	No	151 (47.9)
19. Have you previously attended any session based on concussion awareness?	Yes	0 (0)
	No	315 (100)
20. Will you be willing to attend an awareness campaign for concussion prevention and management?	Yes	211 (67)
	No	104 (33)

Regarding their understanding of concussion, 100% of participants reported prior awareness of the term "concussion." Moreover, 91.7% were also aware of the term "traumatic brain injury," while the remaining 26 (8.3%) were not familiar with the term. Notably, none of the participants (100%) had previously experienced or witnessed traumatic brain injury.

Approximately 68.9% of participants believed that concussion is a type of traumatic brain injury, while 31.1% disagreed. A subset (27.3%) believed that a person could only experience a concussion if they faced a traumatic brain injury resulting in unconsciousness, while 31.7% thought that concussion symptoms always occurred immediately after the injury. In contrast, 72.7% believed that concussion symptoms were not very apparent and might go unnoticed.

Some participants (27.3%) believed that a person could only experience a concussion if they faced a traumatic brain injury resulting in unconsciousness. Additionally, 31.7% thought that concussion symptoms always occurred immediately after the injury, while 72.7% believed that concussion symptoms were not very apparent and might go unnoticed. A total of 31.7% of participants thought that a person without a direct head injury would not experience a concussion, while the remaining 68.3% disagreed.

In terms of reporting concussion symptoms to a doctor, 80.3% believed it was necessary, 13% did not, and 6.7% were unsure. Only 32.4% believed that concussion could not occur years after the injury. About 68.3% of the participants reported that patients affected by concussion should rest and limit both physical and cognitive activities. In contrast, 72.1% believed patients should resume their normal routine after a doctor's advice, and 15.9% thought they should do so after their concussion symptoms had subsided.

Regarding the treatment protocol for concussion, 17.5% considered it straightforward and well-defined, while the majority, 82.5%, believed it was not. In addition, 31.7% thought that all patients with concussion should rest for seven days, whereas 68.3% disagreed. Approximately 29.2% agreed that it was necessary to perform CT scans for all patients with concussion after traumatic brain injury, while 70.8% disagreed. Most participants (85.4%) believed that concussion management was based on symptomatic relief.

Approximately 52.1% felt they had sufficient awareness about concussions. Surprisingly, 315 (100%) of the participants had never attended any sessions on concussion awareness, but 67% expressed their willingness to attend an awareness campaign for concussion prevention and management.

Symptoms of concussion

The most frequently reported symptom of concussion among the participants was a headache (90.5%), followed by changes in vision (74.9%), amnesia (72.1%), difficulty in maintaining body balance (70.5%), disorientation (69.2%), sleep disturbances (62.5%), depression (62.2%), confusion (55.9%), and seizures (42.5%). Mood swings were reported by 25.1% of the participants, while 34% were unsure about the symptoms of concussion.

Sources of information

Participants primarily gained information about concussions from teachers (100%), followed by the internet and social media (81.6%), medical course books (69.8%), family or friends (67.3%), and healthcare providers (24.4%). Only 7.3% mentioned government campaigns as a source of information.

## Discussion

Understanding the level of knowledge and attitudes regarding brain concussion is vital as it shapes the response to this condition and highlights areas where education and awareness can be enhanced [10]. This study aimed to explore the knowledge, attitudes, and behaviors related to concussion among medical students in the Eastern Province of Saudi Arabia, and the findings provide valuable insights.

The demographic profile of the participants reveals a diverse group of medical students. A substantial proportion of the participants were male (56.5%), reflecting the gender distribution within the medical student population. The age distribution was also diverse, with the majority (36.2%) falling within the 23-26 year age group. This age distribution corresponds with the typical age range for medical students. Additionally, the majority of participants reported an average monthly income of less than 10,000 SAR.

The study's mean knowledge score of 24.5 out of 32 suggests that participants displayed varying levels of understanding of concussions. A significant portion (68.3%) demonstrated good knowledge, while 31.7% exhibited poor knowledge.

These findings align with similar studies, such as McKinlay et al. [11], which investigated knowledge levels but targeted the general public and reported that around two-thirds of questions were correctly answered by participants. It is essential to recognize that while a significant proportion demonstrated good knowledge, there is still room for improvement in enhancing overall awareness among the medical student population.

All participants (100%) reported prior awareness of the term "concussion." The majority (91.7%) were also familiar with the term "traumatic brain injury." A noteworthy percentage (68.9%) of participants correctly identified concussions as a type of traumatic brain injury, consistent with the general understanding of concussions. However, it is important to note that the study by Bloodgood et al. [12] reported that only approximately one-quarter of youth and parents demonstrated a basic understanding of concussions, which satisfied specific criteria related to brain injury and symptomatology. This baseline awareness provides a solid foundation for further education and awareness initiatives. It is noteworthy that 72.7% of respondents perceived that concussion symptoms are not always readily apparent and may go unnoticed. This understanding is crucial as it highlights the often subtle nature of concussion symptoms, emphasizing the need for a high index of suspicion when assessing potential cases.

A significant majority (80.3%) believed it was necessary to report concussion symptoms to a doctor, aligning with best practices [3]. This recognition of the importance of professional evaluation is a positive indicator. Additionally, nearly two-thirds (68.3%) agreed that patients affected by concussions should rest and limit both physical and cognitive activities, which is in line with standard recommendations for the initial management of concussions. This knowledge is significant as it indicates an understanding of the need for immediate symptom management.

Participants varied in their opinions on when patients should resume their normal activities. A majority (72.1%) believed that patients should do so only after receiving advice from a doctor, recognizing the importance of medical guidance in post-concussion care. Interestingly, 31.7% of participants believed that all patients with concussions should rest for seven days, while the majority (68.3%) disagreed. This divergence in opinion highlights a specific area for targeted education, emphasizing the individualized nature of concussion management, where a standardized period of rest may not be appropriate for all cases. Furthermore, 29.2% of participants agreed on the necessity of performing CT scans for all patients with concussion after traumatic brain injury, in line with the importance of diagnostic imaging for specific cases. The majority (85.4%) believed that concussion management is centered on symptomatic relief, acknowledging the primary goal of providing comfort and relief to patients. These attitudes align with established clinical principles [3].

Regarding participants' self-assessed awareness levels, approximately 52.1% felt sufficiently informed about concussions. It's worth noting that while over half of the participants expressed confidence in their awareness, knowledge assessment scores varied among the respondents, with 68.3% demonstrating good knowledge about concussions. The willingness to participate in educational initiatives underscores the potential for improving knowledge and awareness through targeted awareness campaigns. It also suggests that there is a demand among medical students for further education on the topic [13].

The most commonly reported symptom of concussion among the participants was a headache (90.5%), followed by changes in vision (74.9%), amnesia (70.5%), and difficulty in maintaining body balance (70.5%). These findings correlate with Broglio et al.'s study [14] and demonstrate an awareness of common concussion symptoms. Recognizing these symptoms is crucial for early identification and management. The substantial percentage of participants expressing uncertainty about concussion symptoms emphasizes the need for enhanced education and awareness initiatives to ensure that individuals, including medical students, have a more comprehensive understanding of the varied symptomatology of concussions.

Participants primarily cited healthcare professionals (100%) as their source of information about concussions, followed by the internet and social media (81.6%). This differs from Beidler et al.'s study, where healthcare providers were the primary information source for most participants [15]. The prevalence of healthcare professionals as a source of information emphasizes the influential role that educators play in shaping students' understanding of medical conditions. This underscores the importance of ensuring that educators themselves have accurate and up-to-date knowledge of concussions. This reliance on the internet and social media as sources of information highlights the need to guide students on how to critically evaluate online sources for reliability and accuracy.

The study presents several limitations that warrant consideration. Firstly, it exclusively focused on medical students from a single medical school in the Eastern Province of Saudi Arabia. While providing valuable insights into this specific group, it may not fully capture the diversity and characteristics of medical students across the entire Eastern Province. Therefore, the generalizability of our findings to a broader population should be approached with caution. Additionally, we have strived to minimize bias in the sampling process and survey distribution. However, inherent limitations and potential sources of bias may persist, which warrant consideration. These limitations underscore the need for future research endeavors to encompass a more diverse participant pool and further investigate potential biases for a more comprehensive understanding of the topic.

## Conclusions

This study conducted among medical students at King Faisal University in the Eastern Province of Saudi Arabia reveals a generally positive level of knowledge and awareness about brain concussions. While the majority of participants demonstrated good knowledge, certain knowledge gaps were identified, particularly in attitudes and procedures related to managing concussions. The findings underscore the importance of continuous education and awareness campaigns to bridge these gaps and ensure that individuals, including medical students, possess a comprehensive understanding of concussions and their management. Additionally, it emphasizes the significant role of healthcare providers in promoting this crucial knowledge.
